# The 14th of April, Past and Present

**DOI:** 10.3390/tropicalmed5020100

**Published:** 2020-06-18

**Authors:** Simone Petraglia Kropf, Nísia Trindade Lima

**Affiliations:** Oswaldo Cruz Foundation (Fiocruz), Rio de Janeiro 21040-360, Brazil; nisia.lima@fiocruz.br

In May 2019, the World Health Organization established the “World Chagas Disease Day”, to be celebrated on the 14th of April. But why choose this date?

Those who are familiar with the history of Chagas disease know that this was the day that Carlos Ribeiro Justiniano Chagas first identified the *Trypanosoma cruzi* infection in a human being, namely the two-year-old girl Berenice Soares de Moura [1,2,3]. Ironically, despite going down in history as the first described case of the new trypanosomiasis, discovered in the hinterlands of Brazil in 1909, she would live for many decades without developing any symptoms of the disease, passing away at 72 years of age due to neurological causes unrelated to Chagas disease.

The landmark discovery, which now frames the “World Chagas Disease Day”, has a significance that goes beyond chronology. Like any memory rite, it recovers and monumentalizes the past from the horizons and perspectives of the present, as well as the future that one wants to project. What, then, is this significance? Why “celebrate” (in the sense of “remembering together”) April 14th?

First, it concerns a celebration of science, by paying homage to a long and successful tradition of research produced in Brazil, that has achieved wide national and international recognition since the beginning [1,3]. It represents clear evidence that Brazilian scientists were, and are, not mere consumers or recipients of theories and ideas coming from European centers, but active subjects (albeit under asymmetric relations) in the production of knowledge [1,3,4,5]. Tropical medicine, of which Chagas disease would become an emblem, went through a decisive moment of institutionalization in the country at the time, which was a direct result of the establishment of the Oswaldo Cruz Institute (the origin of what is now the Oswaldo Cruz Foundation) [3,5,6,7]. It is worth mentioning that this happened only a few years after the creation of the first schools and institutes dedicated to this specialty in Europe.

If the past evoked by memory gains meaning in the present, giving global visibility to this landmark of Brazilian science becomes a political act of affirmation to its importance, not only as an activity that advances the frontiers of knowledge, but as a practice intended to provide solutions to concrete social problems, which requires expertise and actions at local, but also global levels.

In this sense, it projects the value of a scientific tradition deeply committed to the health of historically neglected populations onto the world scene. The science that described a new disease in the small town of Lassance would also describe a different country, the ‘Brazil of the hinterlands’, marked by abandonment and plagued by this and so many other diseases. This was a Brazil quite different from the one that was enjoyed by the elites, who celebrated progress on the French-like avenues in the recently remodeled capital Rio de Janeiro [8]. As Carlos Chagas said since his early work: If Europeans studied tropical diseases because they considered them an obstacle to their colonialist enterprise, studying American trypanosomiasis and other parasitic diseases in Brazil was important because they affected the health of its own populations [9,10]. Understanding and fighting these diseases was, therefore, a central element in a broader project for the construction of a new Brazil [3,8].

This takes us to the second dimension of the significance of the 14th of April: its social meaning, literally embodied in the encounter that took place in 1909. When examining Berenice, who lived in a miserable hut that was infested by triatomine bugs (or ‘kissing bugs’), Carlos Chagas did not just find the parasite he was looking for, but also the tangible and human face of poverty. It was the first of many such faces that he would encounter from then on, which revealed to him the structural “social parasitism” gnawing away at the country through its heritage of colonialism and slavery, as the physician Manoel Bomfim argued in his book “Latin America: evils of origin”, an important work of Brazilian social thought [11].

Carlos Chagas would not tire of saying that disease and poverty were two sides of the same coin, a problem for which the solution depended on the State’s firm action in the implementation of public health policies, aiming to serve Berenice and so many others affected by what he called “diseases of Brazil” [3,5,10,12]. When assuming the leadership of the federal health services ten years later, the scientist from the Oswaldo Cruz Institute would put these ideas into practice by bringing health services and policies to remote corners of Brazil, serving populations that had never seen any sign of public authorities [13]. Therefore, remembering the 14th of April has a political meaning, defending a conception of health as a right of the people and a duty of the State [12].

In the 110 years that have passed since the discovery of Chagas disease, many advances have been made in understanding and coping with this disease, not only in Brazil, but also in other countries [14]. However, despite these advances, the disease still affects an estimated 8 million people worldwide, who, like Berenice, were and are neglected. The biggest challenge, and the main reason for the importance of the 14th of April, is to give visibility to these people, as Carlos Chagas did. But not only that, it is a question of giving them a voice and a leading role, so that they are able to be included as active subjects in the collective undertaking of science and health initiatives aimed at serving them [15].

Experiencing the World Chagas Disease Day amid the COVID-19 pandemic, an emerging disease caused by the coronavirus SARS-CoV-2, gives the date a unique and dramatic tone. May the current health emergency, which mobilizes the world for the imperative need to support science and health, be an occasion to reflect on the structural problems that we have been facing for so many decades as well. May the 14th of April 2020 be an invitation to understand that the path is one: Science in service of health and life, for people with faces and names, like Berenice.
